
JOURNAL INFORMATION
==============================
NLM Title Abbreviation: 3 Biotech
Journal ID: 3 Biotech
NLM Journal ID: 101565857

3 Biotech

ISSN: 2190-572X
EISSN: 2190-5738
Publisher: Springer

ARTICLE INFORMATION
==============================
PMCID: PMC4327753
DOI: 10.1007/s13205-015-0277-6
Article ID: 277
Article version: 1
Subjects: Acknowledgement to Referees

Acknowledgement to Referees

Electronic publication date: 2015 Feb 11
Print publication date: 2015 Feb
Volume: 5
Issue: 1
First page: 107
Last page: 109
Copyright: © The Author(s) 2015
License: This article is distributed under the terms of the Creative Commons Attribution License which permits any use, distribution, and reproduction in any medium, provided the original author(s) and the source are credited.
License URL: https://creativecommons.org/licenses/by/4.0/

issue-copyright-statement: © King Abdulaziz City for Science and Technology 2015

==============================

## Acknowledgement to Referees

The Chief Editors and the Editorial Board thank the reviewers' listed below for their contribution and time consuming efforts in refereeing papers submitted to 3 Biotech in 2014. Their timely response and critical comments have not only assisted us in the selection of high-quality scientific work, but their critique is also appreciated by authors as it frequently assists them to improve the style and content of their publications.

P. Agamuthu, Malaysia
C. Aguilar, Mexico
A. Ahamed, United States
G. Alekhya, India
S. Al-Hajoj, Saudi Arabia
A. Alrajhi, Saudi Arabia
H. Amani, Iran (Islamic Republic Of)
R. Anjum, Germany
C. Arrese-Igor, Spain
U. Avci, United States
G. Babnigg, United States
R.K. Bachheti, Ethiopia
H. Bagheri, Iran (Islamic Republic Of)
D. Barhr, India
P. Basu, United States
M. Baum, United States
K. Bhainsa, India
B. Bharathiraja, India
S. Bhattacharya, India
C. Bi, China
R. Biswas, United States
N. Bozic, Serbia
T. Cai, China
C. Cantacessi, United Kingdom
C. Chakraborty, India
S. Chaluvadi, United States
M.S. Chandra, India
Y. Chao, China
G. Chen, China
W. Chen, China
H. Cheng, China
H. Chopra, India
A. Chow, Canada
R. Chowdhury, India
A. Das, India
A. Datta, United States
D. Dave, Canada
C. de Carvalho, Portugal
I. de la Mata, Spain
M.J. de Lima Brossi, Brazil
B. Delclos, United States
S. Dhanarasu, Saudi Arabia
W. di Iain, Australia
K.K. Doddapaneni, India
P. Dubey, Japan
K. Dutta, India
N. Dzimiri, Saudi Arabia
S. Elchuri, India
C. Engels, Germany
S. Fu, China
R. Galántai, Hungary
H. Gautam, India
R.K. Gawalapu, United States
X. Ge, United States
F. Genta, Brazil
S. Gerdes, United States

H. Ghebeh, Saudi Arabia
D. Ghosh, India
D.D. Giri, India
D. Gokhale, India
K. Goswami, India
S. Gourinath, India
J.R. Gowda, Germany
A. Green, United States
R. Gudihal, India
E. Gundogdu, Turkey
A. Gupta, India
S. Gupta, United States
V. Gupta, India
V. Gupta, United States
M. Hachem, Denmark
Y. Han, China
J.-O. Hoog, Sweden
L. Hou, Japan
Z. Huang, China
S. Ittiyavirah, India
D. Jabbour, United States
M. Jadhav, India
N. Jaiswal, India
M. Jani, India
K.-H. Jhee, Republic of Korea
W. Jiang, China
Y. Jiao, United States
M.C. Jimenez-Martinez, Mexico
M. Jin, United States
S. Johri, India
K. Josyula, United States
K. Kannabiran, India
A. Karthikeyan Adhimoolam, India
R. Kataria, India
K. Kathiresan, India
A. Katiyar, India
M.S. Khan, India
J. Kilbane, United States
W. Klawech, Thailand
A.S. Kumar, India
B. Kumar, India
L. Kumar, India
N. Kumar, India
R. Kumar, United States
V.G. Kumar, India
S. Kushaha, Sweden
R. Lal, India
J. Lemieux, Canada
D. Li, China
T. Li, United States
Y. Li, China
M. Licursi, Argentina
J. Liu, United States
J. Liu, China
L. Liu, China
T. Liu, China
X. Liu, United States
Z. Liu, United States
A. Lo Giudice, Italy
F. Luo, China
J. Luo, United States
Y. Luo, United States
B. Madhesan, United States
N. Mahajan, United States
S.K. Mandal, India
M. Manickavasagam, India
K.S. Manja, India
H. Manonmani, India
V. Megadi, India
S. Mehetre, India
K. Mi, China
S. Middha, India
K. Mirajkar, India
S. Mohsenzadeh, India
A. Mondala, United States
A.K. Mukherjee, India
R. Naidu, Australia
M. Naik, India
S. Nambeesan, United States
C. Nanayakkara, India
R. Nandakumar, India
S. Nandi, India
M. Nassar, Saudi Arabia
N. Nawani, India
T. Nikizaki, Japan
A. Nitschke, Brazil
J. Oakeshott, Australia
L. Pajeva, Bulgaria
P. Palittapongarnpim, Thailand
A. Pandey, India
A. Pandit, India
R. Pandya, India
A.A. Pantazaki, Greece
S.R. Parcha, India
N. Pareek, India
R. Parthasarathi, United States
S.K. Patel, India
P. Peddisetty, India
Y. Peng, China
A. Peralta, United States
P. Piazza, United States
N.R. Prasad, India
B. Prithiviraj, United States
Y. Pu, United States
G. Pusch, United States
Y. Qu, Canada

E.K. Radhakrishnan, India
J.M. Raeed, Bangladesh
A. Rai, India
J. Raj, India
R. Rajasekaran, India
J. Raman, Malaysia
K.R.S.S. Rao, India
S. Ravella, United Kingdom
R.V. Ravishankar, India
R. Ray, India
L.J. Rebecca, India
G. Reddy, India
U.K. Reddy, United States
A. Rehman, Pakistan
H. Reyes-Valdes, Mexico
H. Riveros-Rosas, Mexico
C.A. Rodrigues, Brazil
R. Rodríguez-Sotres, Mexico
J. Ruzicka, Czech Republic
K. Saga, India
D. Saini, India
R. Sandeep, India
A. Sartimbul, United States
M. Sateesh, Republic of Korea
T. Satyanarayana, India
P. Schenk, Australia
S. Seaver, United States
T. Sebastien, New Zealand
P. Seesuriyachan, Thailand
A. Segura, Italy
S.A. Selim, Egypt
G. Selvakumar, India
P. Selvam, India
A. Shanmuganathana, United States
A. Sharma, India
J. Shi, China
A.K. Singh, India
B. Singh, India
H. Singh, India
R. Singh, India
R.S. Singh, India
V. Sivasubramanian, India
S. Smith-Roe, United States
H. Song, China
R. Soni, India
L. Sousa, United States
S. Sriram, India
N. Subbarao, India
K. Tang, Germany
S. Tang, China
A. Teotia, India
A. Thapliyal, India
C.-G. Tian, China
G. Tommonaro, Italy
T. Tsukuba, Japan
R. Tu, China
M. Turan, Turkey
J. Vakhlu, India
K. Velmourougane, India
D. Verma, India
M. Verma, Australia
J. Vicente, United Kingdom
V. Vu, United States
S. Waghmare, India
D. Wang, United States
H. Wang, China
W. Wang, United States
D. Watters, Australia
N. Wei, United States
K.F. Wong, Hong Kong
H. Xiao, China
Z. Xu, China
C.G. Yoo, United States
B. Yu, China
Y. Yuan, United States
D. Zhang, United States
Y. Zhang, China
G.-Q. Zhao, China
Y. Zheng, China
C. Zhou, China
C.L. Zhou, United States
J. Zhou, China
X. Zhou, China
Y. Zhou, Australia
K. Zhu, China
X. Zhu, China
Y. Zhu, China

Open Access

This article is distributed under the terms of the Creative Commons Attribution License which permits any use, distribution, and reproduction in any medium, provided the original author(s) and the source are credited.

